# Benchmarking of Decellularization Protocols for Small Intestinal Submucosa: Defining the Gold Standard for Functional Tissue Engineering

**DOI:** 10.3390/ma19091803

**Published:** 2026-04-28

**Authors:** Carlos Adams, Iván Gómez, Odin Rodríguez, Alexey Vigil, Cecilio Hernández, Jorge Ceballos, Bruno A. Cisterna, Diego Reginensi

**Affiliations:** 1 Regenerative Therapies, Faculty of Biosciences and Public Health, Universidad Especializada de las Américas (UDELAS), Panama City 0849-0141, Panama; carlossadams7@gmail.com (C.A.); ivan200316@gmail.com (I.G.); rodriguezodin18@gmail.com (O.R.); 2 Regenerative Therapies, Interdisciplinary Center for Research in Life and Health Sciences (CIVSAL), Faculty of Health Science and Engineering, Universidad Latina de Panama (ULATINA), Panama City 0849-0141, Panama; alexeyvigil27@gmail.com; 3 Doctorate Program in Biosciences and Biotechnology, Faculty of Sciences and Technology, Universidad Tecnológica de Panama (UTP), Panama City 0849-0141, Panama; cecilio.hernandez@utp.ac.pa (C.H.); jorge.ceballos1@utp.ac.pa (J.C.); 4 Department of Neuroscience and Regenerative Medicine, Medical College of Georgia, Augusta University, Augusta, GA 30912, USA; bcisternairrazab@augusta.edu; 5 Center for Biodiversity and Drug Discovery, Institute of Scientific Research and High Technology Services of Panama (INDICASAT-AIP), City of Knowledge, Panama City 0843-01103, Panama

**Keywords:** Small Intestinal Submucosa (SIS), decellularization strategy, ECM scaffold, biomimetic materials, skin repair, wound healing, tissue engineering

## Abstract

**Highlights:**

**What are the main findings?**

- Four published SIS decellularization protocols representing detergent-based, chaotropic-salt-based, and sequential ionic/alkaline–acidic strategies were benchmarked.
- The identification of the optimal decellularization protocol according to molecular, structural, and biomechanical parameters.
- The selected decellularized SIS scaffold was well tolerated in a pilot test using a murine acute wound model and showed early tissue integration.

**What are the implications of the main findings?**

- A strategy type has a major impact on the final quality of SIS-derived scaffolds.
- A benchmarking framework based on molecular, structural, and mechanical criteria can support the development of SIS scaffolds for skin applications.

**Abstract:**

The fabrication of decellularized small intestinal submucosa (dSIS) requires a precise balance between effective cellular removal and the preservation of structural integrity. In this study, we compared four published dSIS protocols representing detergent-based, chaotropic-salt-based, and sequential ionic/alkaline–acidic strategies. Their performance was evaluated based on residual DNA, collagen preservation, surface ultrastructure, and mechanical properties. The best decellularization protocol demonstrated the lowest residual DNA levels, together with better collagen retention, scaffold architecture, and mechanical performance than the other methods tested. The selected decellularized scaffold was used in a murine acute wound model and showed good biocompatibility and integration with the surrounding tissue at 10 days after implantation. However, further extensive testing in murine models is essential before future scaling. Finally, this comparative study provides a practical framework for selecting dSIS preparation methods for skin repair applications.

## 1. Introduction

Traditional approaches, including saline-based care, passive dressings, and advanced wound management strategies such as synthetic materials, dermal substitutes, and negative-pressure wound therapy, are often insufficient for the treatment of complex wounds [1,2,3]. For this reason, new strategies derived from tissue engineering [4,5,6] and modern nanomedicine [7,8,9] are emerging as cutting-edge treatments in biomedicine, highlighting advanced biomaterials, such as bioactive dressings [10,11] and decellularized tissues [12,13,14,15], that show promising results. However, these scaffolds are not commonly used in hospital medical applications [16,17].

Because of this, there is increasing interest in biomaterials that can provide more than simple wound coverage. Decellularized small intestinal submucosa (dSIS) supports skin lesion healing by serving as a scaffold that promotes re-epithelialization, angiogenesis, and modulation of inflammation. It can support a pro-regenerative microenvironment and offers cytocompatibility, high bioactivity, and suitable mechanical properties for tissue regeneration [18]. dSIS promotes regeneration by reducing destructive enzymes, maintaining growth factors (FGF-2, TGF-β, VEGF), shifting immune response from M1 to M2, and containing matrix-bound nanovesicles (MBVs) [19,20,21], and offering mechanical properties that serve as a template for tissue growth with natural 3D architecture, adaptability, and fluid management [19,22].

Compared with other decellularized matrices such as dermis, pericardium, or urinary bladder matrix (UBM), dSIS has been reported to display distinct biological and structural features, including intrinsic bioactivity, accelerated degradation kinetics that enable constructive remodeling and structural adaptability, and M2 macrophage polarization. Additionally, dSIS shows more uniform thickness and mechanical consistency, facilitating reproducibility [19,23,24,25]. Despite many investigations into dSIS production, its clinical use is not yet established. This highlights the need for rigorous studies to evaluate biofabrication protocols [26,27]. To address this gap, we conducted a comparative analysis of leading SIS decellularization protocols to elucidate the fabrication process for these bioactive scaffolds.

The quality of a decellularized scaffold depends strongly on how the tissue is processed. A protocol that efficiently removes cellular material may also alter the matrix, whereas a milder approach may better preserve structure but leave more residual material. Different decellularization strategies may therefore vary in their ability to remove cellular material while preserving the structural and mechanical features required for scaffold function. For that reason, direct comparison of decellularization methods is important when the goal is to obtain a scaffold with useful biological and mechanical properties [28,29,30].

In this study, we compared four published porcine SIS decellularization protocols representing three different processing strategies: detergent-based, chaotropic-salt-based, and sequential ionic/alkaline–acidic approaches [31,32,33,34]. Our goal was to determine which strategy provided the most favorable overall balance between effective decellularization and preservation of scaffold quality. For this comparison, benchmarking was based on three levels: (i) quantitative biological criteria, specifically residual DNA content <50 ng/mg and fragments <200 bp, (ii) preservation of structural tissue (e.g., SEM, collagen levels) and (iii) maintenance of biomechanical properties, such as ultimate tensile strength (UTS), elasticity, and Young’s modulus [28,29,30].

As a fundamental starting point, we conducted a simple compatibility study of the patch with a murine model. These initial tests on acute injuries serve as a crucial bridge for the transition from in vitro trials to in vivo application. This pilot study anchors our systematic evaluation, which is crucial for resolving the dilemma of translating advanced skin scaffolds into clinical practice and for finding the right balance among the immune response, functional integrity, and mechanical properties of dSIS-based biomaterials [32,33,34].

## 2. Materials and Methods

### 2.1. Isolation the Small Intestinal Submucosa (SIS)

Porcine small intestines were obtained from a municipal slaughterhouse (Panama City, Panama) through a strictly regulated process, with attention to animal welfare (3Rs). The tissue was transferred under strict sterile conditions, maintaining the cold chain. In a laminar flow hood, the jejunum was dissected into 10 cm longitudinal segments. After cutting, the segments were immersed in a decontamination solution containing 1% penicillin and streptomycin (Sigma-Aldrich, St. Louis, MO, USA, Cat. # A5955-100 mL) and 0.1% peracetic acid (Sigma-Aldrich, Cat. # 269336-100 mL). After decontamination, the small intestinal sections were rinsed eight times with 1× PBS for 2 min per wash to remove residual reagents. The mucosal layer was removed with a surgical scalpel, followed by careful removal of the serosal and muscular layers with ligature forceps, leaving the submucosal layer intact. Finally, the SIS was stored at −80 °C to preserve its structure and prevent DNA degradation [35,36,37].

### 2.2. Decellularization of Small Intestinal Submucosa (SIS)

Four published SIS decellularization protocols, representing three processing strategies, were evaluated to generate decellularized small intestinal submucosa (dSIS) scaffolds. For clarity, these methods are referred to throughout the manuscript as protocols A–D. Protocols A and B were detergent-based approaches, protocol C was a sequential ionic/alkaline–acidic approach, and protocol D was a chaotropic-salt-based approach [31,32,33,34]. These strategies differed in chemical composition, exposure conditions, and total processing time. The associated protocols are detailed below:

- Protocol A (Detergent combination, short time), based on Rashtbar et al. (2018) [33], SIS samples were incubated in phosphate-buffered saline (PBS) (Thomas Scientific, Chadds Ford Township, PA, USA, Cat. # P3813) supplemented with 1% penicillin and streptomycin for 48 h at 37 °C. Decellularization was performed with 0.05% sodium dodecyl sulfate (SDS; IBI Scientific, Cat. # IB070062) for 6 h, followed by 0.1% Triton X-100 (VWR, Cat. # M143-1L) for 3 h at 37 °C. Ionic detergents and cellular debris were removed through three 30 min washes in distilled water. The total process takes about 66 h (roughly 2.75 days).
- Protocol B (Detergent combination, long time), described by Duong et al. (2024) [32], SIS samples were treated with 1% SDS for 72 h, followed by 10% Triton X-100 for 24 h. Residual detergents were removed by washing with deionized water for 72 h. All procedures were performed at room temperature (RT). The total processing time was 168 h (7 days).
- Protocol C (Ionic/alkaline–acidic): As indicated by Abraham et al. (2000) [31], SIS samples were incubated in PBS containing ethylenediaminetetraacetic acid (EDTA) (Sigma-Aldrich, Cat. # E9884-500G) at 100 mM and sodium hydroxide (NaOH) (EMD Millipore, Burlington, MA, USA, Cat. # 1.06498.1000) at 10 mM at pH 11 for 16 h. Samples were then incubated in 1 M hydrochloric acid (HCl) (Millipore, Cat. # 1003172500) and 1 M sodium chloride (NaCl) (Sigma-Aldrich, Cat. # S5886-500G) in 1× PBS at pH 1 for 8 h. Afterward, the samples were neutralized for 16 h with 1 M sodium chloride (NaCl) at pH 7.4, followed by three 30 min washes in 1× PBS and one final 2 h wash in distilled water. All processes were carried out at R.T., and the total processing time was 48 h (2 days).
- Protocol D (Chaotropic salt): As described by Singh et al. (2022) [34], SIS samples were incubated in 1M potassium iodide (KI) and then in 0.1% Triton X-100 for 24 h. Samples were then washed three times for 30 min in sterile distilled water. All processes were performed at R.T., and the total processing time was 48 h (2 days).

All decellularization processes were performed under mechanical agitation at 90 rpm on an orbital shaker (VWR Incu-shaker Mini, Benchmark).

### 2.3. Post-Processing of Decellularized Small Intestinal Submucosa (dSIS)

After decellularization, SIS tissues were lyophilized and sterilized with UV light for 30 min (15 min per side). The lyophilized samples were stored at −80 °C [30], and the non-lyophilized samples were stored at 4 °C [34].

### 2.4. Qualitative and Quantitative Analysis of Decellularized Small Intestinal Submucosa (dSIS)

#### 2.4.1. DNA Isolation of dSIS

For DNA extraction, 1 mg of lyophilized tissue was resuspended in lysis buffer (1% SDS, 100 mM NaCl, 10 mM Tris, 25 mM EDTA, and 0.1 mg/mL proteinase K) and incubated at 37 °C for 60 h. DNA was extracted with phenol:chloroform:isoamyl alcohol (25:24:1). The aqueous phase was recovered and precipitated with ice-cold 3.0 M sodium acetate (0.1 volume) and 100% ethanol (2.5 volumes). The DNA pellet was washed with 70% ethanol, centrifuged, and air-dried. The DNA pellet was reconstituted in nuclease-free water. Samples were stored at −20 °C until subsequent analysis [14,34,38].

#### 2.4.2. Agarose Gel Electrophoresis of dSIS

To assess DNA fragment size, 5 µL of each DNA sample was mixed with 1 µL of loading dye and loaded onto an agarose gel together with a molecular weight ladder. Electrophoresis was performed in 1X TBE buffer (Research Products International, Cat. # IB01020) at a constant voltage of 80–120 V for 30–60 min. DNA bands were visualized under UV light using a transilluminator (VWR, Cat. # 76407-432), and a digital image was used to analyze fragment distribution [34,38].

#### 2.4.3. DNA Fluorometric Quantification of dSIS

Residual DNA concentration was quantified using the Quant-iT™ PicoGreen™ dsDNA Assay Kit (Thermo Scientific, Waltham, MA, USA, Cat. # P11496) according to the manufacturer’s instructions. The PicoGreen reagent was diluted 1:200 in TE buffer to prepare the working solution. A standard curve was generated using standard lambda DNA (2 µg/mL in TE buffer) by serial dilutions from 25 pg/mL to 1000 ng/mL. A final volume of 100 µL of the standard solutions and our samples was loaded into a 96-well microplate, and 100 µL of PicoGreen reagent (diluted 1:200) was added to each well. After 5 min of incubation in the dark at room temperature, fluorescence was measured at 480/520 nm using a BioTek Synergy H1 multimode reader [14,34].

### 2.5. Collagen Quantification of Decellularized Small Intestinal Submucosa (dSIS)

Total collagen content was measured using the Sircol Soluble Collagen Assay (Biocolor, Belfast, UK, Cat. # S1000) according to the manufacturer’s instructions, with minor modifications. Briefly, dSIS samples (20 mg) were digested in a solution of 0.1 mg/mL pepsin and 0.5 mg/mL acetic acid for 24 min with constant stirring at room temperature. After centrifugation at 3000× *g* for 10 min, the supernatant was collected and mixed with Sircol dye reagent for 30 min. The collagen–dye complex was pelleted by centrifugation at 12,000× *g* for 10 min, washed in acid-saline reagent, centrifuged again, and finally dissolved in alkaline reagent. Samples were transferred to a 96-well plate, and absorbance was read at 562 nm using a microplate reader (ACURIS, Denver, CO, USA, SmartReader™ 96 Absorbance Plate Reader) [39,40].

### 2.6. Ultrastructural Analysis of Decellularized Small Intestinal Submucosa (dSIS)

For scanning electron microscopy (SEM), samples were fixed in a standard Karnovsky solution (2% paraformaldehyde, 2.5% glutaraldehyde, and 0.1 M phosphate buffer) for 90 min at room temperature. Samples then underwent sequential, ascending-gradient dehydration with ethanol (30%, 50%, 70%, 80%, 90%, and 100% *v*/*v*), with each step carried out for 10 min at room temperature. After dehydration, specimens were critical-point dried (Leica, EM CPD300) and sputter-coated with palladium–gold (VacTechnique DSR1). Ultrastructural images were acquired using a Zeiss Evo 10 scanning electron microscope (SEM) at the Universidad Tecnológica de Panamá, Panamá, at an accelerating voltage of 15 kV [33,38,41]. Surface analysis of the samples was performed using the ImageJ v1.54r, Surface Plot plugin, which provides a three-dimensional visualization in which pixel positions (X, Y) define the base, and pixel brightness (pixel value) represents the height (Z) [42].

### 2.7. Mechanical Characterization of Decellularized Small Intestinal Submucosa (dSIS)

Mechanical properties were evaluated using a uniaxial tensile test on a Universal Tensile Testing Machine (Shimadzu, Model AGS-J). The objective of this biomechanical study was to determine the ultimate tensile strength (UTS), elongation, and E-modulus (Young’s modulus) for each decellularized intestinal tissue obtained from the four protocols. Samples were cut into rectangular shapes (10 mm × 5 mm) and stored in 1× PBS until testing. Each sample was mounted between the grips of the testing machine and stretched at a displacement rate of 10 mm/min until failure. Stress–strain curves were recorded and used to calculate UTS, elongation at break, and Young’s modulus for each protocol [33,34,43].

### 2.8. Pilot Biocompatibility Assessment in a Murine Acute Wound Model

Biocompatibility was evaluated in a pilot testing assay in a murine acute wound model using female C57BL/6 mice aged 7–9 weeks and weighing 19–25 g. Female mice were chosen because they exhibit lower aggression during housing [44,45]. Before surgery, ciprofloxacin (30 mg/kg) was administered to prevent infection; meloxicam (5 mg/kg, SC) was used as an analgesic; and physiological saline was injected subcutaneously to prevent dehydration during the procedure [46].

Anesthesia was induced with 1–2% isoflurane and confirmed by loss of reflexes (e.g., paw and tail reflexes) [47,48]. The mice were placed in sternal recumbency to apply ophthalmic gel (SAVAL, Nico-tears gel) and to prevent corneal dryness and ulcers [49]. After, the mouse’s back was shaved and disinfected with 2% chlorhexidine gluconate, a broad-spectrum antiseptic. An 8 mm circular wound was then made along the dorsal midline using a biological punch and surgical scissors [50,51]. For the scaffold group, the dSIS was implanted and sutured into the wound bed, aligned with the tissue margins. A four- or five-strand non-absorbable nylon suture (Ethilon 5/0) was used to attach the scaffold to the mouse skin, keeping the edges slightly above the biomaterial [46,51]. Additionally, a transparent film dressing (Tegaderm) was applied over the wound for protection throughout the 10-day experimental period. At the end of the 10-day experimental period, mice were euthanized via isoflurane inhalation, in strict accordance with institutional animal care guidelines and established ethical protocols.

This pilot study bridges the in vitro to in vivo gap by evaluating the material integrity of the skin patch and its compatibility with the biological tissue. This ensures ethical 3R compliance and refines experimental parameters before large-scale validation. For the skin wound experiments, a total of 12 mice were used and were divided into two subgroups: group A (negative control) had wounds without a biomaterial (*n* = 6), and group B (decellularized scaffold) had wounds treated with a decellularized biomaterial (*n* = 6). Mice were housed individually to prevent physical contact, such as biting or scratching, and to reduce stress. The study was conducted at the INDICASAT-AIP animal facility.

## 3. Results

### 3.1. Obtaining the Porcine Small Intestinal Submucosa (SIS)

**Figure 1:**
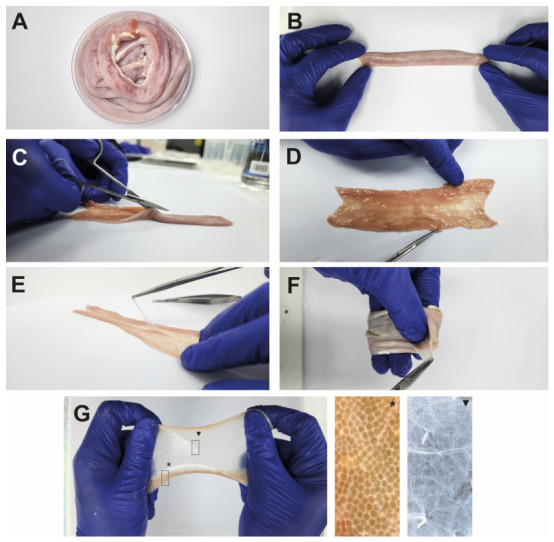
**Workflow for obtaining small intestinal submucosa (SIS).** (**A**) Macroscopic view of native jejunum under sterile conditions. (**B**) Manual extension of the native jejunum. (**C**) Longitudinal incision of the intestinal tissue. (**D**) Flat orientation for mechanical dissection. (**E**) Removal of the tunica serosa, muscularis externa, and mucosa layers for isolation of the SIS. (**F**) Flexibility and handling properties of the SIS. (**G**) SIS under tension, exhibits elastic deformation. The asterisk (*) indicates collagen fibers, and the triangle (▼) identifies the elastic fibers.

The native jejunal tissue exhibited intense pink coloration, visible vasculature, and preserved gross morphology. Mechanical delamination was used to remove the serosa and external muscular layers, followed by removal of the inner luminal mucosal layer, yielding isolated SIS. Macroscopic analysis of the SIS revealed a thin, uniform structure and a translucent, pale-yellow appearance. These macroscopic features indicated that the manual dissection procedure was effective for isolating the submucosal layer used for scaffold preparation (Figure 1).

### 3.2. Obtaining Decellularized Small Intestinal Submucosa (dSIS)

**Figure 2:**
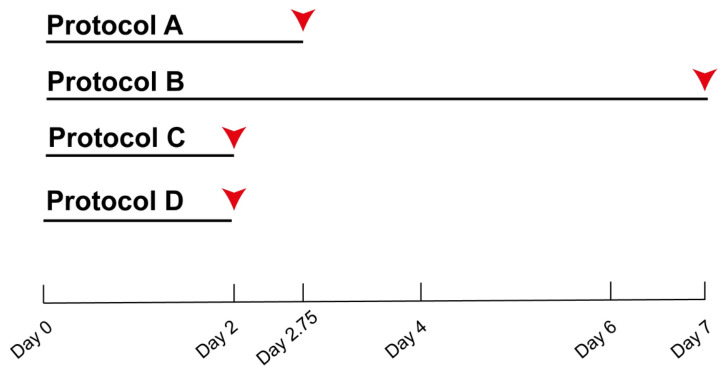
**Comparative timeline of the four small intestinal submucosa (SIS) decellularization protocols.** Protocols C and D were completed in 48 h (2 days). Protocol A required 66 h (2.75 days), and Protocol B was the longest, requiring 168 h (7 days).

Processing time was an important point of comparison among the decellularization protocols. A first point of comparison among the four decellularization methods, which represented three different processing strategies, was total processing time. The results showed that two protocols, C and D, were completed in 48 h (2 days); Protocol A took slightly longer, at 66 h (2.5 days); and Protocol B was the most time-consuming, requiring 168 h (7 days) (Figure 2).

**Figure 3:**
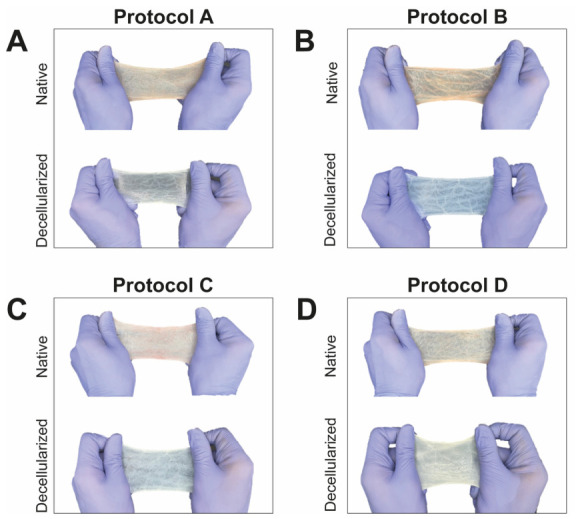
**Macroscopic appearance of the small intestinal submucosa (SIS) before and after decellularization.** Native SIS and scaffolds produced using protocols (**A**–**D**) are shown under resting and manual tension conditions. Changes in color, translucency, and gross integrity are shown for qualitative comparison.

Native SIS tissue exhibits an opaque yellow color, characteristic of cellular material and residual blood components. The results obtained after the application of each of the four evaluation protocols are described below, highlighting aspects of visual structure and simple manual stretching (Figure 3):

- Protocol A showed tissue discoloration after decellularization. Furthermore, physical and structural changes occur in the tissue due to the combination of detergents, both ionic (e.g., SDS) and non-ionic (e.g., Triton X-100), which are capable of solubilizing cell membranes and denaturing various proteins, leading to damage to the tissue fibers (e.g., collagen, elastin). The duration of this protocol was 66 h (Figure 3A).
- Protocol B showed significant pigmentation loss and a translucent appearance, indicating complete decellularization. However, prolonged exposure caused structural deterioration and damage to the scaffold architecture. The total duration of this protocol was 168 h (Figure 3B).
- Protocol C showed a transition from an opaque yellowish color (native) to a translucent-appearing tissue (decellularized), reflecting successful decellularization. Furthermore, excellent structural preservation of the scaffold was achieved, including preservation of longitudinal elastic fibers that provide tensile strength. The total duration of this protocol was 48 h (Figure 3C).
- Protocol D showed that the transition from an opaque yellowish color to a translucent-appearing tissue can be observed, indicating macroscopic partial decellularization. Additionally, excellent structural preservation of the scaffold was achieved, with retention of fibers and tissue topography. The total duration of this protocol was 48 h (Figure 3D).

### 3.3. Qualitative and Quantitative DNA Analysis of Decellularized Small Intestinal Submucosa (dSIS)

**Figure 4:**
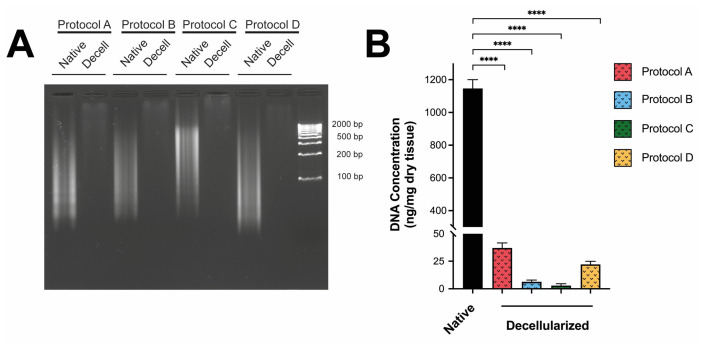
**Validation of decellularization efficacy by quantitative and qualitative DNA analysis.** (**A**) Agarose gel electrophoresis showing high-molecular-weight DNA in native SIS and a marked reduction in visible DNA signal in decellularized groups. (**B**) Residual DNA quantification (ng/mg dry weight) in native and decellularized SIS. The dashed line indicates the commonly accepted threshold of <50 ng/mg dry tissue. Data are presented as mean ± SD (*n* = 3). Statistical significance is indicated by asterisks (****, *p* < 0.0001).

DNA integrity was first assessed by agarose gel electrophoresis. Native SIS showed an intense, diffuse band extending up to 2000 bp, indicating the amount of intact genomic DNA in the original tissue. All decellularization protocols showed high efficiency and the near-complete disappearance of DNA bands. None of the four protocols produced fragments smaller than 200 bp, which is the criterion for successful decellularization (Figure 4A).

Quantitative analysis of residual DNA content in decellularized samples was performed using the fluorometric assay to evaluate the effectiveness of the different decellularization protocols. DNA quantification showed that native intestinal submucosal tissue had a high genetic material content, averaging approximately 1146.26 ng/mg dry weight. All four decellularization protocols reduced DNA levels below 50 ng/mg, the established decellularization criterion [52]. Protocols B and C achieved the greatest decellularization, with residual DNA levels of 6.67 and 2.98 ng/mg dry weight, respectively, while protocols A (25.11 ng/mg dry weight) and D (32.48 ng/mg dry weight) showed slightly higher values (Figure 4B). Together, these results show that all protocols achieved substantial DNA removal, with protocol C showing the strongest performance in this part of the analysis.

### 3.4. Analysis of the Surface Ultrastructure of Decellularized Small Intestinal Submucosa (dSIS)

**Figure 5:**
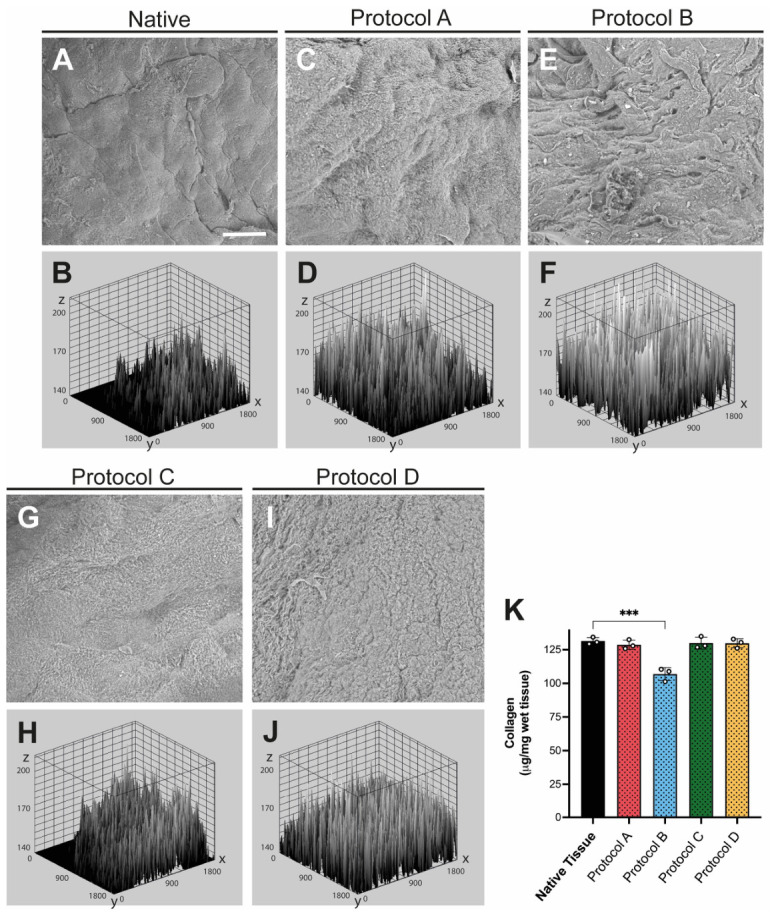
**Surface ultrastructure and collagen content of decellularized small intestinal submucosa (dSIS).** (**A**,**C**,**E**,**G**,**I**) Representative scanning electron microscopy (SEM) images of native SIS and scaffolds generated using protocols A–D. (**B**,**D**,**F**,**H**,**J**) Corresponding surface plot reconstructions derived from the SEM images. (**A**,**B**) Native SIS. (**C**,**D**) Protocol A, (**E**,**F**) Protocol B, (**G**,**H**) Protocol C, and (**I**,**J**) Protocol D. (**K**) Analysis of collagen levels in native and decellularized tissue. Scale bar: 25 μm. The data are presented as mean ± SD (*n* = 3). Statistical significance is indicated by asterisks (***, *p* < 0.001).

SEM analysis revealed clear differences in surface morphology across all the protocols. The native SIS showed grooves and ridges, confirming successful preservation of the ECM. In the surface plot, the native tissue exhibited moderate peaks and somewhat uneven topography (Figure 5A,B). For Protocol A, the onset of mild-to-moderate structural alteration was observed. This was evidenced by irregular indentations, deeper cavities, and a rougher, more cracked surface, indicating early architectural disruption and topographic changes (Figure 5C,D). For Protocol B, the most severe structural alteration (severe damage) was observed, with marked loss of the original surface organization and a more granular appearance. The plot graph indicated extensive erosion of the surface layer (Figure 5E,F). Protocols C and D showed intermediate changes, with visible surface irregularities but better preservation of overall scaffold organization than Protocol B. The surface plot showed a dense and relatively homogeneous distribution of peaks, consistent with preservation of surface topography (Figure 5G–J). Collagen quantification showed that native SIS had the highest collagen content, approximately 130 µg/mg dry tissue. Protocols A, C, and D yielded results similar to native SIS, around 130 µg/mg, indicating that most collagen was preserved, whereas Protocol B showed a decrease, dropping to levels close to 100 µg/mg dry tissue (Figure 5K).

Taken together, these findings indicate that protocols A, C, and D preserved collagen better than protocol B, while the combination of SEM and collagen analysis placed protocol C among the best-preserved scaffolds in this comparison. The combination of total collagen quantification with scanning electron microscopy (SEM) was used to establish a direct link between biochemical composition and microstructural organization. While total collagen concentration serves as the primary biomarker for ECM preservation after decellularization, SEM allows visualization of whether this collagen is properly assembled into fibers, the degree of their alignment, and the presence of porosity or degradation. This dual approach is critical for confirming both the biochemical and structural preservation of a mechanically competent fibrillar network.

### 3.5. Mechanical Properties of Decellularized Small Intestinal Submucosa (dSIS)

Mechanical testing showed differences among the four decellularization protocols in tensile strength, elasticity, and stiffness. Protocol C had the highest tensile strength (4.10 MPa), and Protocol D had the lowest (1.52 MPa). Protocols C and D were the most elastic, indicating the preservation of elastic structures. Young’s modulus (E-modulus) measures resistance to elastic deformation. Protocol C had an E-modulus of 20.11 MPa, whereas Protocols A and D showed lower stiffness (Table 1).

Protocol C produced a decellularized scaffold with a robust structure in only 2 days. The dSIS had significantly less DNA and retained much collagen. Additionally, it showed the best mechanical performance and was the most robust of the four protocols. In terms of processing time, macroscopic appearance, residual DNA removal, collagen preservation, ultrastructural topography, and mechanical data, Protocol C showed the most favorable overall performance among the four methods evaluated. Protocol C was selected for in vivo evaluation because it has an elastic modulus similar to that of skin, high structural integrity, and a high UTS, thereby preventing tearing during suture placement (Table 2).

### 3.6. dSIS Biocompatibility Pilot Testing Assay in Murine Model

**Figure 6:**
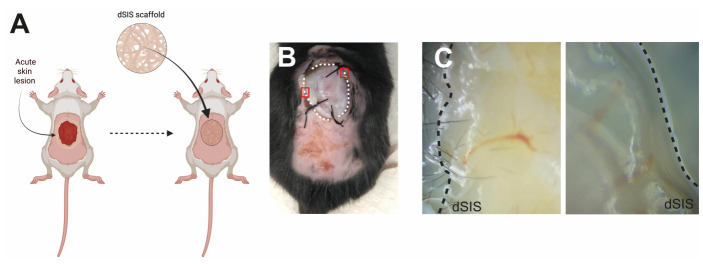
**In vivo pilot assessment of the integration of decellularized intestinal submucosal scaffolds (dSIS) in a murine acute wound model.** (**A**) Schematic representation of wound creation and scaffold implantation. (**B**) Representative macroscopic view of the implantation site. The dotted line indicates the area of the biomaterial secured by sutures. (**C**) Magnified view (Red Box) of the interface between the implanted scaffold (dSIS) and surrounding tissue 10 days after implantation.

The in vivo response to the dSIS scaffold selected from Protocol C (sequential ionic/alkaline–acidic strategy) was evaluated in a pilot study using a murine acute wound model. The scaffold-treated group showed good local integration with the surrounding tissue at the implantation site. The dSIS appeared well integrated with the surrounding host tissue 10 days after surgery. Together, these findings support the biocompatibility of the selected scaffold and its ability to integrate with host tissue in an acute wound setting (Figure 6).

## 4. Discussion

SIS has been widely studied in regenerative medicine because it can provide more than passive coverage. As a decellularized extracellular matrix, SIS retains structural features of the native tissue and may also preserve matrix-associated signals that support repair, which makes it relevant for wound-related applications [12,19,21,27]. The evaluation of SIS decellularization protocols seeks a “favorable balance” among the removal of cellular components, preservation of bioactive molecules, and maintenance of biophysical properties [52,53].

In the present study, the goal was not only to compare four published protocols but also to examine three different decellularization strategies for porcine SIS, namely detergent-based, chaotropic-salt-based, and sequential ionic/alkaline–acidic approaches [31,32,33,34]. This distinction is important because the quality of a decellularized scaffold depends on more than one outcome, and a method that removes cellular material efficiently does not necessarily preserve the matrix well enough for downstream use [28,29,30].

One of the main findings of this work is that the best-performing scaffold was not defined by DNA removal alone. All four protocols reduced residual DNA below the commonly accepted threshold for decellularized tissues, indicating that each strategy was capable of substantial cellular clearance [28,30,54]. However, important differences appeared once collagen retention, surface ultrastructure, mechanical behavior, and processing time were considered together. In this study, Protocol C produced the lowest residual DNA values while also maintaining collagen content close to native SIS, preserving scaffold morphology better than the harsher treatment, and exhibiting the strongest mechanical performance among the decellularized groups. These results suggest that, under the conditions tested here, this strategy limited the trade-off between effective decellularization and matrix damage more successfully than the other methods [29,30,31,32,33,34].

The comparison between strategies also helps explain why protocol B was less favorable overall despite strong DNA removal. Both detergent-based approaches reduced cellular material efficiently, but the more aggressive detergent conditions and longer exposure used in protocol B were associated with greater structural alteration, lower collagen retention, and the longest processing time. This pattern is consistent with the general concern that stronger decellularization treatments may improve tissue clearance at the expense of extracellular matrix preservation [28,29,30]. By contrast, the chaotropic-salt-based strategy showed intermediate performance, with acceptable decellularization and reasonable preservation of gross scaffold features, but without surpassing the sequential ionic/alkaline–acidic approach in the combined analysis. Taken together, these findings support the idea that comparing strategy types is more informative than focusing on any single protocol component in isolation.

Total collagen was used as a practical indicator of matrix preservation, while SEM provided a side-by-side view of surface organization after decellularization. These measurements do not fully resolve the extracellular matrix composition, collagen subtype distribution, crosslinking state, or the retention of other matrix-associated molecules [19,22,28,30]. In this study, Protocol C exhibits a collagen density and an intact ultrastructure, as observed by SEM, that mimic the biochemical composition and guiding architecture of the human dermis [55,56,57], and mechanical properties closely align with the physiological range of native skin, which reports strengths of 1–20 MPa and elastic moduli of 4.2–38.5 MPa [58,59,60]. These structural and mechanical features support the potential relevance of this scaffold for skin repair applications.

The in vivo experiment should be viewed in the same careful way. The scaffold selected from Protocol C (sequential ionic/alkaline–acidic strategy) was evaluated in a murine acute wound model and showed biocompatibility and local integration at the implantation site after 10 days. These findings are encouraging because they suggest that the material can be implanted and tolerated in a wound environment [35,49,51]. At the same time, this was an acute model with a relatively early endpoint, so the present study does not establish long-term remodeling, full functional repair, or performance in chronic wounds. For this reason, the data from the pilot study of cytocompatibility of the decellularized scaffold are a preliminary stage, for future studies with more complete murine models (e.g., vascularization, immune response) and their possible translation in the future into biomedical applications.

Currently, dSIS is a versatile and promising therapy for skin wounds, hernia repair, and urological, cardiovascular, and gastrointestinal surgeries [61,62]. Despite the abundant supply of source tissue and the logistical feasibility of large-scale dSIS implementation, a gap remains between experimental development and broader clinical translation [18,63,64]. Commercial products based on dSIS, such as Oasis Wound Matrix and Cook Biodesign [65,66,67], have been validated in clinical uses like managing chronic wounds—diabetic foot ulcers, venous leg ulcers, pressure injuries, and reconstructive surgery—by offering bioactive and mechanical properties that enhance healing [68,69]. dSIS commercial products provide better structural support, improved biointegration, and eliminate donor-site morbidity compared with conventional treatments such as synthetic scaffolds and autologous transplants. dSIS is a viable alternative for large-scale, widespread application in the care of complex wounds [70,71]. The standardization and reproducibility of the optimized protocol suggest that the resulting decellularized scaffold is fully suitable for industrial-scale production, offering a cost-effective, open-access solution for generating an advanced scaffold for treating complex pathologies (such as diabetic foot ulcers, pressure ulcers, and reconstructive surgeries).

Overall, the present results support the use of a multi-parameter benchmarking strategy when comparing SIS decellularization methods. Residual DNA alone would not have been sufficient to identify the most suitable scaffold, because the strongest candidate was the one that combined effective cell removal with better preservation of collagen, scaffold morphology, and mechanical strength [29,30,54]. Under the conditions tested here, Protocol C provided a more consistent balance than the detergent-based or chaotropic-salt-based strategies. Although additional work will be needed to expand matrix characterization and evaluate longer-term in vivo outcomes, these data provide a practical basis for selecting this approach for the preparation of SIS-based scaffolds intended for skin repair applications [12,21]. The integration of established laboratory technologies and optimization under rigorous scientific standards facilitates the validation and confirmation that dSIS is an exemplary biomedical application capable of bridging the gap between a validated laboratory concept and a scalable clinical application.

## 5. Conclusions

This study provides a comparative evaluation of four published protocols for porcine SIS decellularization, encompassing three distinct processing strategies, and shows that scaffold quality is best assessed through a multi-parameter framework rather than DNA removal alone. Although all four methods reduced residual DNA below the accepted threshold for decellularized tissues, they differed substantially in collagen retention, surface ultrastructure, mechanical behavior, and processing time. Among the approaches examined, the sequential ionic/alkaline–acidic strategy offered the most favorable overall balance between efficient decellularization and preservation of scaffold integrity. Under the conditions tested here, Protocol C therefore emerged as the most suitable approach for the preparation of SIS-based scaffolds intended for skin repair applications.

The selected scaffold also demonstrated good short-term biocompatibility and local integration in a murine acute wound model, supporting its potential relevance for further preclinical evaluation. While these in vivo findings remain preliminary, they indicate that the optimized dSIS scaffold can be implanted and tolerated in a biologically relevant wound setting.

Overall, these findings establish a practical benchmarking basis for the evaluation of SIS decellularization methods and support the further development of Protocol C as a promising strategy for the generation of skin-relevant biomaterials. Future work should expand matrix characterization and assess longer-term remodeling, immune response, and functional repair outcomes in order to better define the translational potential of this scaffold platform.

## Figures and Tables

**Table 1:** **Mechanical properties of decellularized small intestinal submucosa (dSIS) obtained using Protocols A–D.** Tensile parameters were evaluated from uniaxial testing of native and decellularized samples (Protocols A–D). Parameters include: (i) ultimate tensile strength (UTS), (ii) elongation at break, and (iii) apparent elastic modulus (E-modulus). Cross-sectional area measurements used for stress calculations are also provided. The data are presented as mean ± SD (*n* = 3).

	Native	Protocol A	Protocol B	Protocol C	Protocol D
**UTS (MPa)**	2.78 ± 0.30	1.18 ± 0.37	1.78 ± 0.44	4.10 ± 0.86	1.52 ± 0.42
**Elongation (mm)**	16.97 ± 1.80	17.95 ± 3.33	12.10 ± 2.12	22.39 ± 3.90	24.88 ± 4.53
**E- modulus (MPa)**	29.50 ± 1.60	4.10 ± 0.72	8.81 ± 0.70	20.11 ± 3.07	6.34 ± 1.49
**Cross-sectional área (mm^2^)**	4.28 ± 0.90	4.02 ± 0.50	4.05 ± 0.40	3.67 ± 1.41	4.45 ± 1.15

**Table 2:** **Comparative summary of the four SIS decellularization protocols.** A summary of five variables was analyzed for decellularization protocols (Protocols A-D): (i) Protocol efficiency: decellularization execution time, (ii) Macroscopic analysis: visual integrity of the biomaterial, (iii) Decellularization success: removal of genetic material from the scaffold, (iv) Ultrastructural profile: topographic analysis of the decellularized scaffold using ultrastructural techniques, and (v) Mechanical profiling: a complete analysis of the mechanical properties of the dSIS scaffold. The symbols indicate: + (low to moderate performance), ++ (moderate performance), +++ (high performance), and ++++ (very high performance).

	Time Protocol	Visual ECM Integrity	DNA Removal	Surface Topography	Biomechanics Properties
**Protocol A**	++++	++++	+++	++++	+++
**Protocol B**	++	+++	++++	+	+
**Protocol C**	++++	++++	++++	++++	++++
**Protocol D**	++++	++++	+++	+++	++

## Data Availability

The original contributions presented in this study are included in the article. Further inquiries can be directed to the corresponding author.

## Abbreviations

The following abbreviations are used in this manuscript:

dSIS	Decellularized Small Intestinal Submucosa
ECM	Extracellular Matrix
dECM	Decellularized Extracellular Matrix
VEGF	Vascular Endothelial Growth Factor
FGF	Fibroblast Growth Factor
TGF-β	Transforming Growth Factor β
